# Supplementary material for: Structural basis for mutually exclusive co-transcriptional nuclear cap-binding complexes with either NELF-E or ARS2
Source: Nat Commun. 2017 Nov 3;8:1302. doi: 10.1038/s41467-017-01402-w (PMC5670239; doi:10.1038/s41467-017-01402-w)
Supplement: Supplementary file 1 — Supplementary Information [file 41467_2017_1402_MOESM1_ESM.pdf]

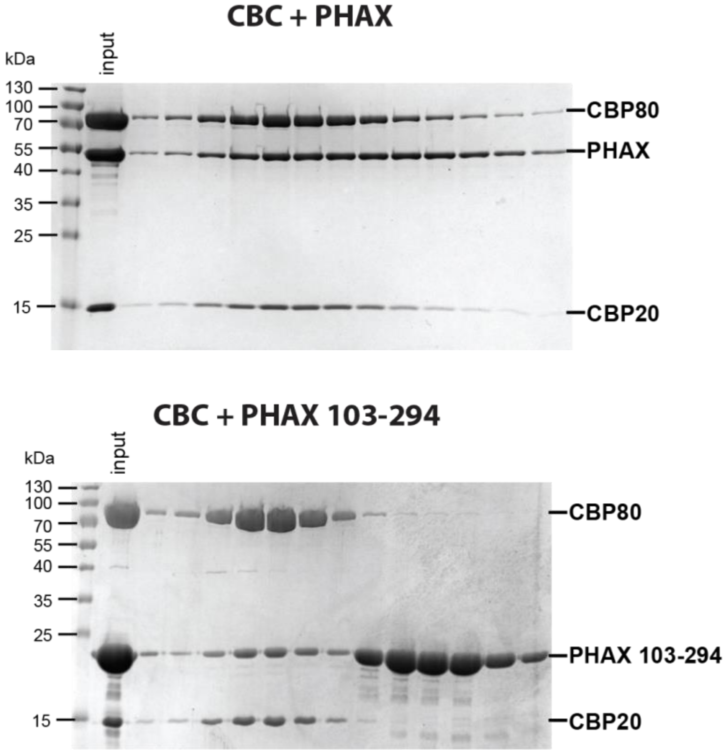

Supplementary Figure 1. Binding of full-length PHAX$^{1\text{-}394}$ and minimal variant PHAX$^{103\text{-}294}$ to CBC as assayed by SEC.

Reconstitution of the CBC-PHAX complex. Purified recombinant CBC and PHAX or PHAX$^{103\text{-}294}$ were mixed and subjected to size exclusion chromatography. The input and the protein concentrating fractions were analysed by Coomassie-stained SDS-PAGE.

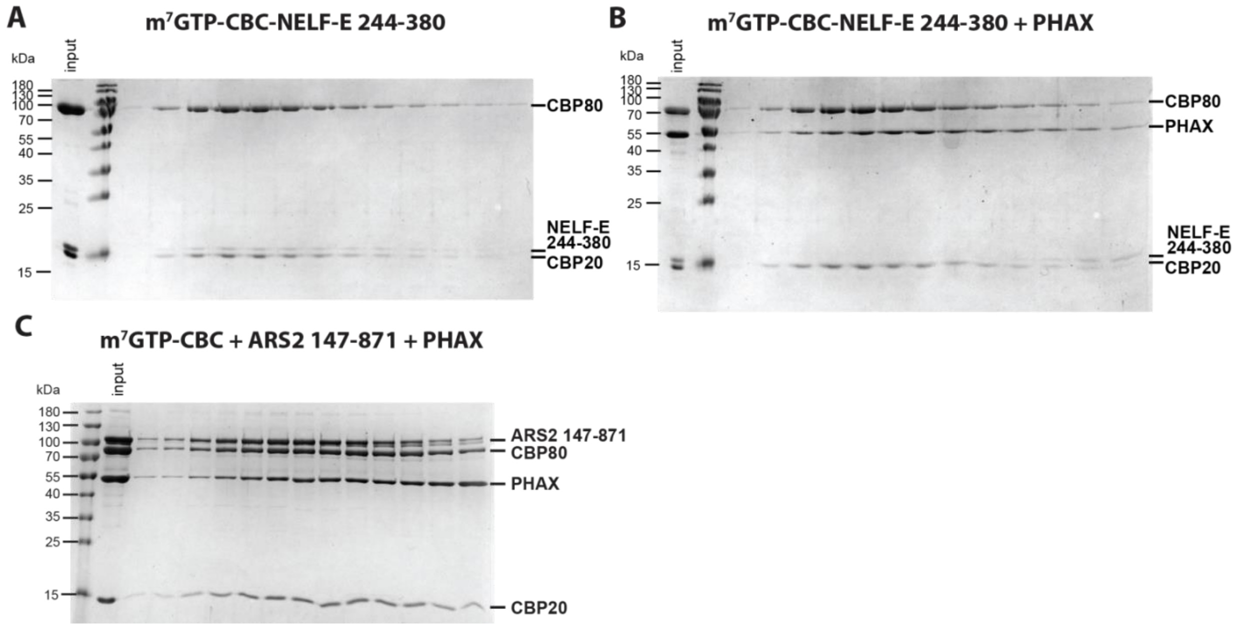

Supplementary Figure 2. **PHAX binding to CBC is compatible with ARS2 binding but not with NELF-E binding.**

A: Coomassie-stained SDS-PAGE from the gel filtration fractions of the reconstituted m^7GTP-CBC-NELF-$E^{244-380}$ complex.

B: Coomassie-stained SDS-PAGE from the gel filtration fractions of the reconstituted m^7GTP-CBC-NELF-$E^{244-380}$ complex mixed with 2x molar excess of PHAX before injection.

C: Coomassie-stained SDS-PAGE of the reconstitution of the m^7GTP-CBC-$ARS2^{147-871}$-PHAX complex by size exclusion chromatography. Purified recombinant CBC, $ARS2^{147-871}$ and PHAX were mixed with m^7GTP and subjected to gel filtration and the single fractions were analysed by SDS-PAGE.

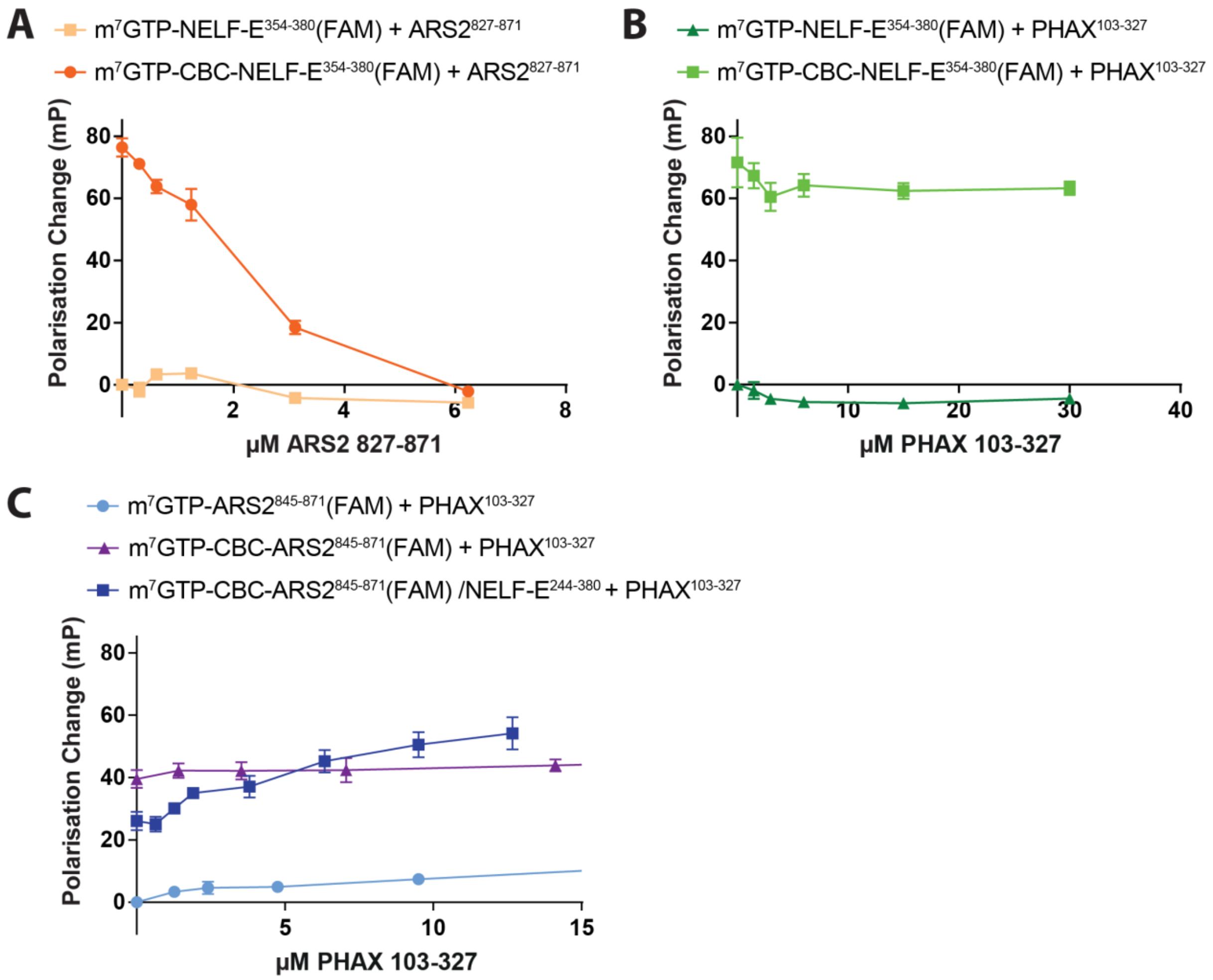

Supplementary Figure 3. Fluorescence polarisation experiments with controls.

To exclude interactions between FAM-ARS2$^{827-871}$ and PHAX or NELF-E, or between FAM-NELF-E$^{354-380}$ and ARS2 or PHAX, control experiments without CBC were performed. To validate the competition between PHAX and NELF-E, PHAX was titrated to a preformed m^{7}GTP-CBC- ARS2$^{845-871}$(FAM) complex. Error bars show the SD of three experiments.

A: ARS2$^{827-871}$ titrated to m^{7}GTP/NELF-E$^{354-380}$(FAM) and to m^{7}GTP-CBC- NELF-E$^{354-380}$(FAM).

B: PHAX$^{103-327}$ titrated to m^{7}GTP/NELF-E$^{354-380}$(FAM) and to m^{7}GTP-CBC- NELF-E$^{354-380}$(FAM).

C: PHAX$^{103-327}$ titrated to m^{7}GTP/ARS2$^{845-871}$(FAM), to m^{7}GTP-CBC- ARS2$^{845-871}$(FAM) and to m^{7}GTP-CBC-NELF-E$^{244-380}$/ ARS2$^{845-871}$(FAM).

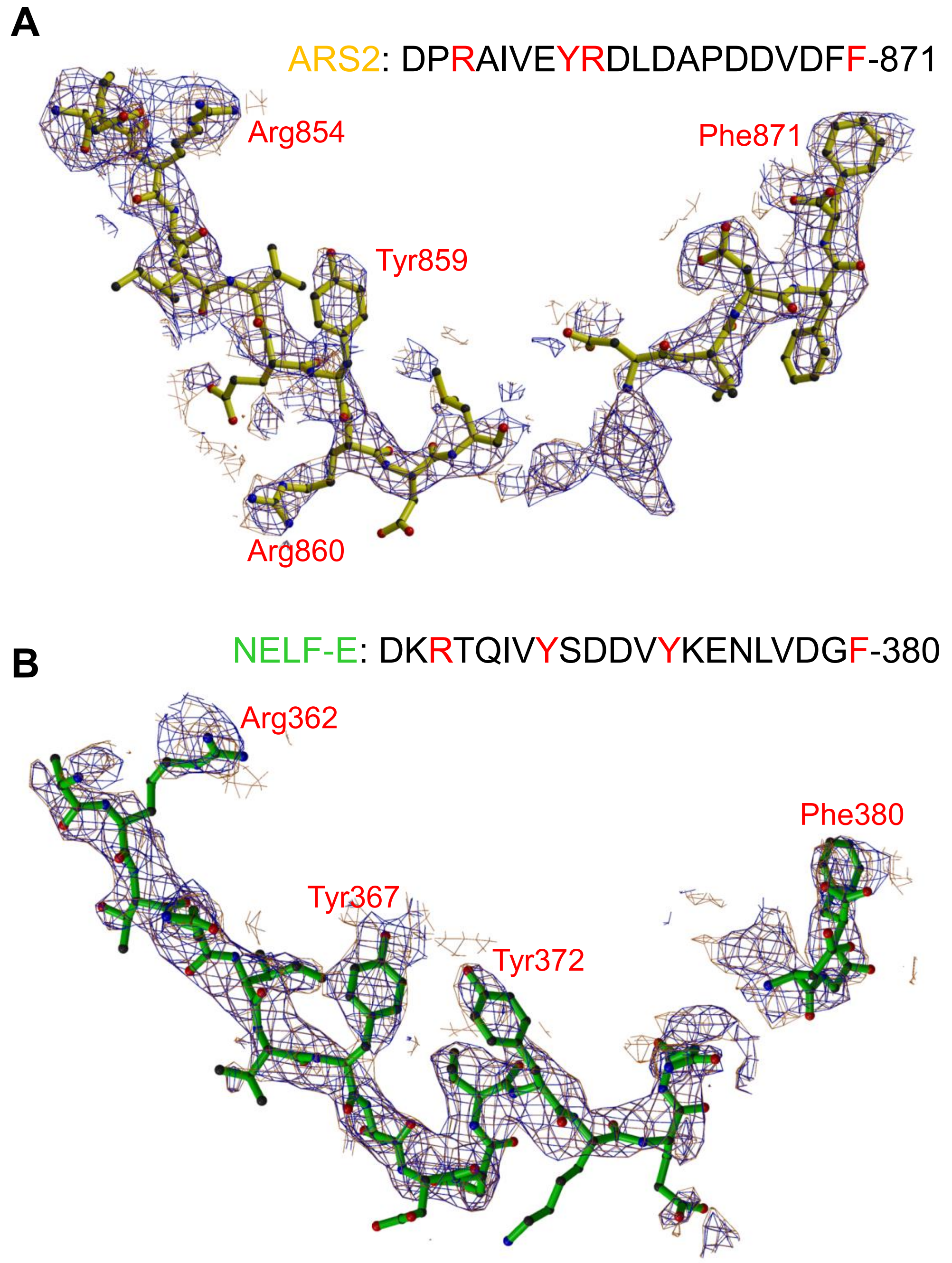

Supplementary Figure 4. Electron density for ARS2 and NELF-E C-terminal peptides bound to CBC. Drawn with BOBSCRIPT[1].

A: Omit difference electron density for residues 852-871 of the C-terminal ARS2 peptide (chain O), contoured at 2 σ.

B: Omit difference electron density for NELF-E$^{360-380}$ C-terminal peptide (chain E), contoured at 2 σ.

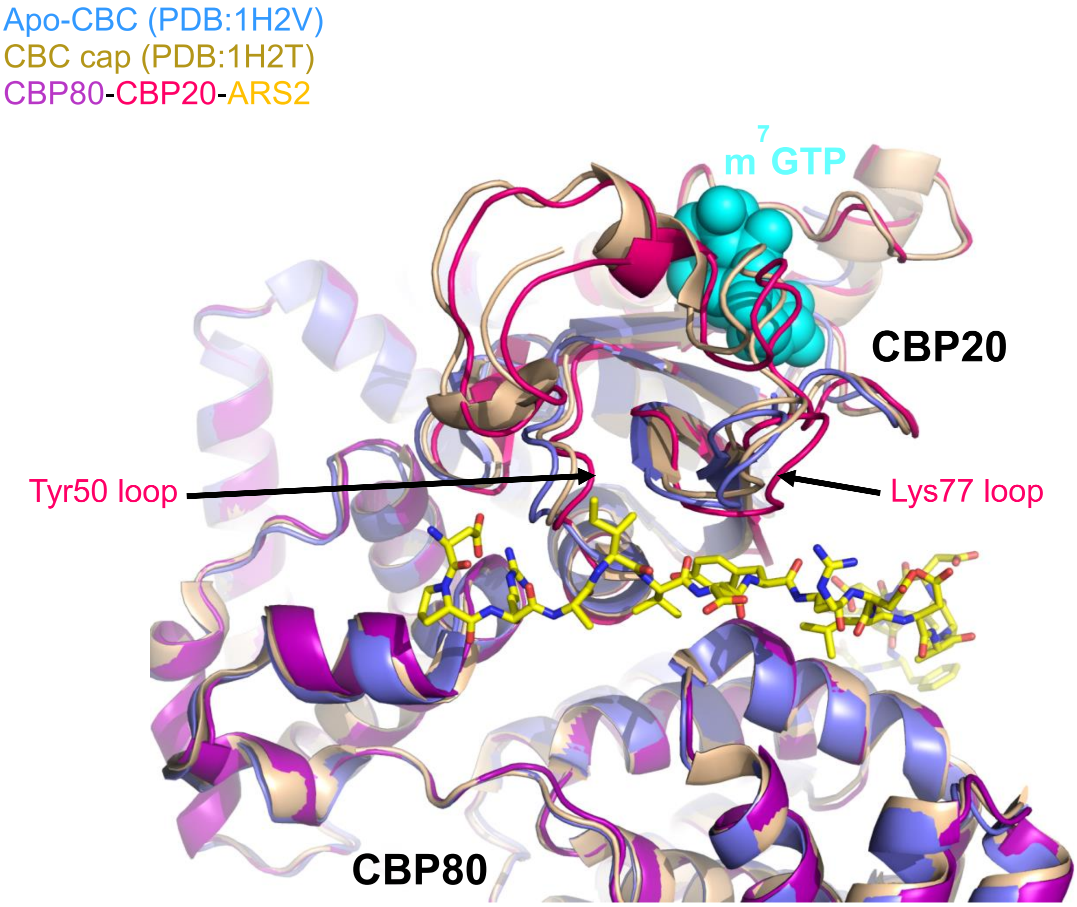

Supplementary Figure 5. Changes in the CBP20-CBP80 interface upon ligand binding. Structural alignment via the CBP80 subunit of apo-CBC (PDB:1H2V, blue), CBC-m^{7}GpppG complex (PDB:1H2T, sand) and m^{7}GTP-CBC-ARS2 complex (CBP20 pink, CBP80 purple, ARS2 yellow, m^{7}GTP cyan) shows successive relative displacements of the CBP20 subunit. Note that much of CBP20 is disordered in the absence of bound cap. In the m^{7}GTP-CBC-ARS2 complex the maximal displacements of CBP20 occur in the Tyr50 and Lys77 loops (labelled, see text).

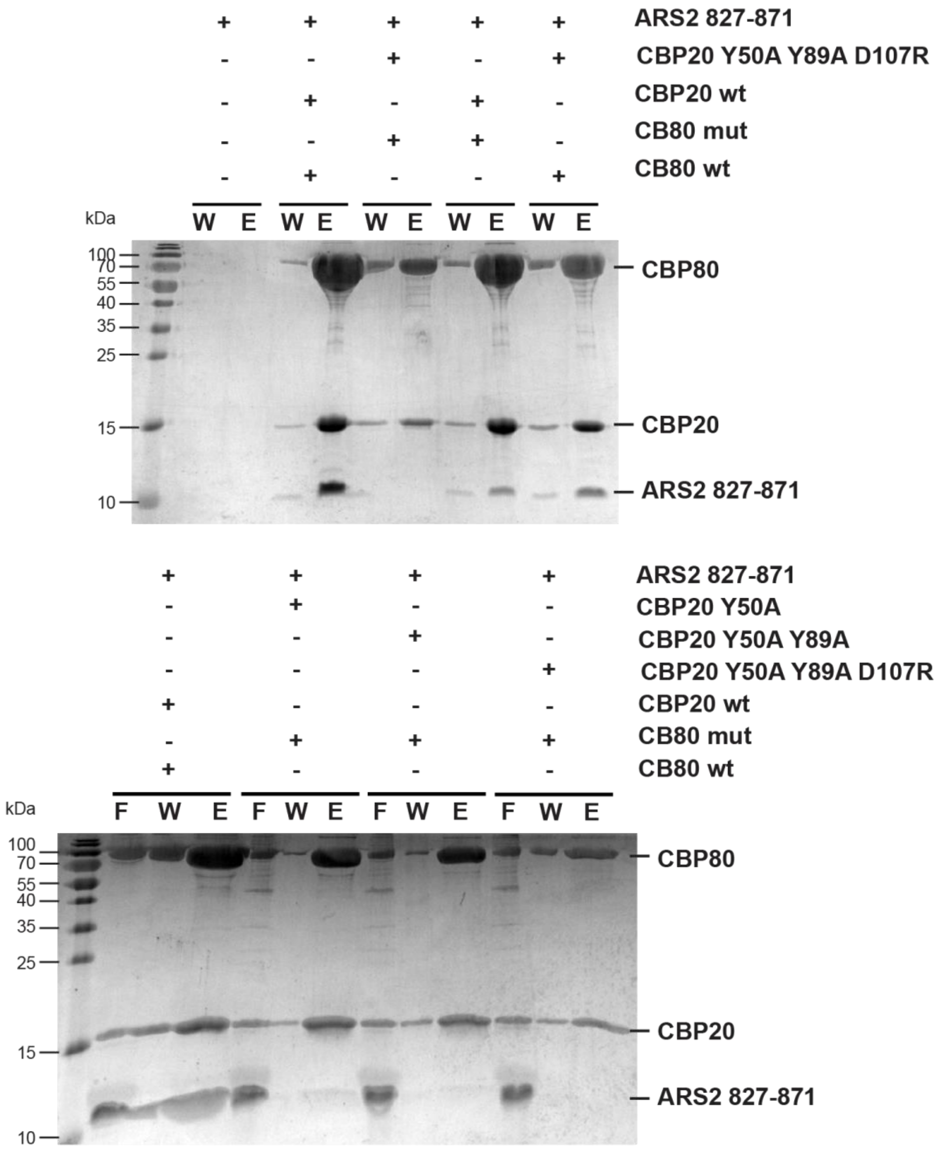

Supplementary Figure 6. Analysis of the interaction between CBC mutants and ARS2$^{827-871}$. CBC was reconstituted with various combinations of mutations in CBP20 and/or CBP80 as indicated and immobilised on m^{7}GTP-sepharose. After incubation with ARS2$^{827-871}$ the washed (W) and elution (E) fractions were analysed by SDS-PAGE followed by Coomassie staining. The lower gel additionally shows the flow through (F). Note that CBC mut combines the single Y50A mutation in CBP20 together with the CBP80 triple mutation Y461A/R610E/H651A (CBP80 mut).

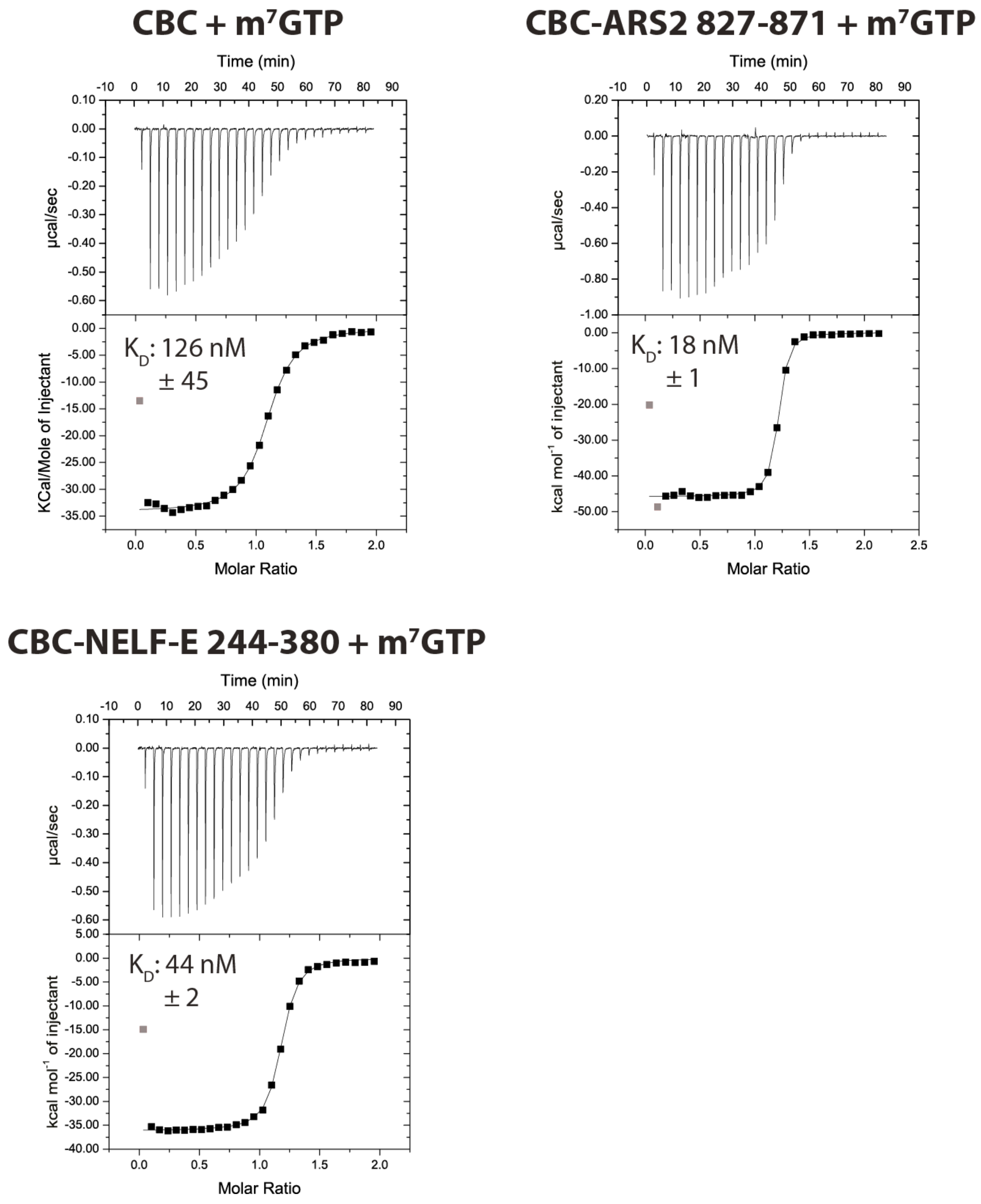

Supplementary Figure 7. Influence of ARS2 and NELF-E on m^7GTP binding to CBC.

Representative isothermal titration calorimetry data, presented as in the caption to Figure 1C, allowing determination of the affinity of CBC to m^7GTP in the absence (left) or in the presence of bound $ARS2^{827-871}$ (middle) or NELF-$E^{244-380}$ (right). CBC or CBC saturated with 2.2 molar excess of $ARS2^{827-871}$ or NELF-$E^{244-380}$ in the sample cell was titrated by m^7GTP. K_D values represent the average from at least two independent experiments (see Table 1).

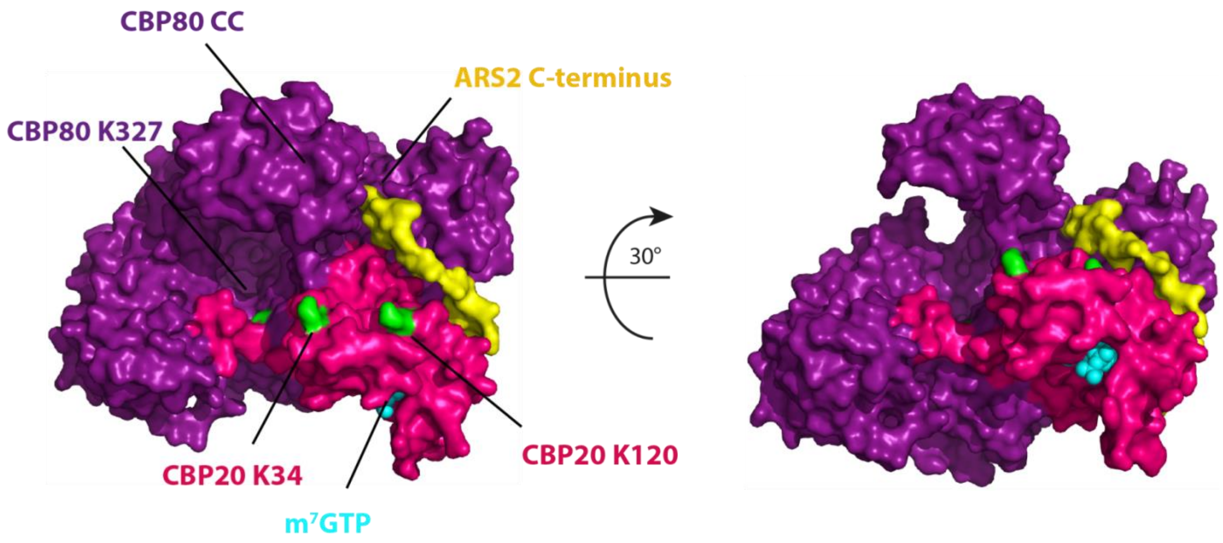

Supplementary Figure 8. **Putative PHAX binding site mapped on to the m^{7}GTP-CBC-ARS2 structure.** Two views of the location of the cross-linked CBC lysines (green) identified within the CBC-PHAX complex (Figure 3B) projected onto the surface of the m^{7}GTP-CBC-ARS2 structure (CBP80 purple, CBP20 pink, ARS2 yellow, m^{7}GTP cyan).

Supplementary Table 1. Inter- and intra-protein cross-links identified within the *in vitro* reconstituted CBC-PHAX complex. Cross-linked lysines are underlined. The ld (linear discriminant) confidence scores were calculated by xQuest[2]. The cross-links were deemed highly confident if their ld score was > 28.

Cross-linked peptide sequences (inter-protein)	protein1	protein2	AA 1	AA 2	ld-Score
NHPQMIAVLVDKMIR-TDWDAGFKEGR	CBP80	CBP20	607	120	36.51
LGNRPEMNYKGR-TDWDAGFKEGR	PHAX	CBP20	216	120	35.25
KESQEHTK-SHWKER	PHAX	CBP80	162	327	34.91
HVLKIQK-SGDIKK	CBP80	CBP20	654	67	34.57
KESQEHTK-SHWKER	PHAX	CBP80	162	327	34.1
NHPQMIAVLVDKMIR-KIIMGLDK	CBP80	CBP20	607	68	33.35
KESQEHTK-SHWKER	PHAX	CBP80	162	327	32.24
KESQEHTK-SHWKER	PHAX	CBP80	162	327	31.6
TDWDAGFKEGR-SHWKER	CBP20	CBP80	120	327	31.03
TDWDAGFKEGR-SHWKER	CBP20	CBP80	120	327	30.84
TDWDAGFKEGR-IIGNKK	CBP20	PHAX	120	256	30.03
DLDKELDEYMHGGKK-GDNEEQEKLLK	PHAX	CBP20	173	34	28.13

Cross-linked peptide sequences (intra-protein)	protein	AA 1	AA 2	ld-Score
LQEKVESAQSEQK-HVLKIQK	CBP80	698	654	42.69
YEITAEDSQEKVADEISFR-KDLIAR	PHAX	229	243	42.27
SSDRKDGVLEEQIER-ELEEAKEKLAR	CBP80	684	665	38.82
LQEKVESAQSEQK-HVLKIQK	CBP80	698	654	38.13
QSETYNYLLAKKLR-KESQEHTK	PHAX	158	162	37.76
YEITAEDSQEKVADEISFR-KDLIAR	PHAX	229	243	37.57
YEITAEDSQEKVADEISFR-KDLIAR	PHAX	229	243	37.48
IQKELEEAK-KMNKHVL	CBP80	657	650	36.49
LQEKVESAQSEQK-HVLKIQK	CBP80	698	654	36.34
LGNRPEMNYKGR-KDLIAR	PHAX	216	243	35.81
TQVLGKK-MKQAIK	PHAX	332	335	35.42
LGNRPEMNYKGR-KDLIAR	PHAX	216	243	34.71
LGNRPEMNYKGR-KESQEHTK	PHAX	216	162	34.44
TQVLGKK-MKQAIK	PHAX	332	335	34.39
YEITAEDSQEKVADEISFR-LQEPKK	PHAX	229	242	34.1
IQKELEEAKEK-MNKHVLK	CBP80	657	654	33.97
GRYEITAEDSQEKVADEISFR-KDLIAR	PHAX	229	243	29.05
GDNEEQEKLLK-TDWDAGFKEGR	CBP20	34	120	29.03
KESQEHTK-IIGNKK	PHAX	162	256	29.01

Supplementary Table 2. Primers used in this work.

Primer	Sequence 5' to 3'
**PHAX**	
PHAX fw (NcoI)	CTACGCCATGGCGTTGGAGGTCGGCGAT
PHAX rev (HindIII)	ACGCTAAGCTTTTAAAAGATGTCCAAATC
PHAX 327 rev (XhoI)	TCTCGAGTTATTGTGTGTTCTCCTCTTCCT
PHAX 294 rev (XhoI)	GAATCTCGAGCTATTTCAAGAGATTCAGAAAAACTCC
PHAX 264 rev (XhoI)	GAATCTCGAGCTATTCCATCAGAAGTTCAATTGC
PHAX 308 rev (XhoI)	GAATCTCGAGCTAGAAAATGTCCTTAATTTGTTCCTCG
PHAX 120 fw (NcoI)	CATGCCATG GCTGTGCTGCAGGAACAG
**NELF-E**	
NELF-E fw (NcoI)	CTTTATTTTCAGGGCGCCATGTTGGTGATACCCCCCGGAC
NELF-E 244 fw (NcoI)	CTTTATTTTCAGGGCGCCATGGGTCCTTTCCGCAGGTCGG
NELF-E rev (XhoI)	GGTGGTGGTGGTGCTCGAGTCAGAAGCCATCCACAAGGTTTTCC
NELF-E 360 rev (XhoI)	GGTGGTGGTGGTGCTCGAGTCAGTCCCGGTGGCAACCCTTAGG
**ARS2**	
ARS2 147 fw (NcoI)	CATGCCATGGGCCCCGTGATGAAGACCTTCAAGGAGT
ARS2 763 fw (NcoI)	CATGCCATGGAGATCAAGCCAGCCCAGC
ARS2 827 fw (NcoI)	CATGCCATGGGT CGA GGG AAC TAT GAT GC
ARS2 845 rev (XhoI)	GAATCTCGAGTCAGCGAGGTTTCCCAGGAT
ARS2 rev (XhoI)	GAATCTCGGAGTCAAAAGAAATCAACATCGT
**CBP20**	
CBP20 fw (NcoI)	CTTTATTTTCAGGGCGCCATGGAATGTCGGGTGGCCTCCTGAAG
CBP20 rev (XhoI)	GAATCTCGGAGTCAGGAAAACTGGCACAGAACCAG
**Mutagenesis**	
CBP20 Y50A fw	ATCTTTCTTTTGCCACAACTGAAGAACAAATC
CBP20 Y50A rev	TCTTCAGTTGTGGCAAAAGAAAGATTTCCAAC
CBP20 D107R fw	ATTCGCACACGCTGGGACGCAGGCTTTAAGG

CBP20 D107R rev	AGCCTGCGTCCCAGCGTGTGCGAATGATTCG
CBP20 Y89A fw	TTGTGGAATATGCCTCACGCGCAGATGCG
CBP20 Y89A rev	TCTGCGCGTGAGGCATATTCCACAAAACAG
CBP80 Y461A fw	AGGTTGTCTGCCCATCAGCGTATATTAGATAT
CBP80 Y461A rev	TACGCTGATGGGCAGACAACCTCATACATTTTTC
CBP80 R610E fw	AAGATGATTGAGACACAAATAGTTGATTGTGC
CBP80 R610E rev	AACTATTTGTGTCTCAATCATCTTATCCAC
CBP80 H651A fw	ATGAACAAAGCTGTCCTGAAGATCCAGAAAGAG
CBP80 H651A rev	ATCTTCAGGACAGCTTTGTTCATCTTACG
ARS2 R854A Y859A fw	GCGGCCATTGTGGAAGCTCGGGACCTGGATGCCC
ARS2 R854A Y859A rev	GCTTCCACAATGGCCGCTGGGTCTCCACGAACCATCC
ARS2 F871D fw	ATGTTGATTTCGATTGACTCGAGCACCACCACCACC
ARS2 F871D rev	TGCTCGAGTCAATCGAAATCAACATCGTCTGG GGCATCC

Supplementary References.

1. Esnouf RM. Further additions to Molscript version 1.4, including reading and contouring of electron density maps. *Acta Crystallogr* **55**, 938-940 (1999).

2. Leitner A, Walzthoeni T, Aebersold R. Lysine-specific chemical cross-linking of protein complexes and identification of cross-linking sites using LC-MS/MS and the xQuest/xProphet software pipeline. *Nature protocols* **9**, 120-137 (2014).
